# Professor Conway

**Published:** 1865-02

**Authors:** 


*Medical College of Virginia.*

The Board of Visitors of this Institution will convene at the College Building, in Richmond, on Wednesday, April 5th, 1865, to fill the Chair of Obstetrics and Diseases of Women and Children, made vacant by the death of Professor Conway.

All communications on this subject should be addressed to the President of the Board of Visitors of the Medical College of Virginia, care of Professor L. S. Joynes, Dean of the Faculty.

